# Effect of Baseline Characteristics on Cabazitaxel Treatment Duration in Patients with Metastatic Castration-Resistant Prostate Cancer: A Post Hoc Analysis of the Compassionate Use/Expanded Access Programs and CAPRISTANA Registry

**DOI:** 10.3390/cancers12040995

**Published:** 2020-04-17

**Authors:** Zafar Malik, Giuseppe Di Lorenzo, Angelika Pichler, Ugo De Giorgi, Simon Hitier, Evelyne Ecstein-Fraisse, Ayse Ozatilgan, Joan Carles

**Affiliations:** 1 Clinical Oncology, The Clatterbridge Cancer Centre NHS Foundation Trust, Wirral CH63 4JY, UK; 2 Department of Medicine and Health Sciences ‘Vincenzo Tiberio’, University of Molise, 86100 Campobasso, Italy; 3 Medical Oncology, Tortora Hospital, 84016 Pagani, Salerno, Italy; 4 Department of Hematology and Oncology, Regional Hospital Hochsteiermark, 8700 Leoben, Austria; 5 Istituto Scientifico Romagnolo per lo Studio e la Cura dei Tumori (IRST) IRCCS, 47014 Meldola, Italy; 6 Department of Biostatistics, Sanofi, 91380 Chilly-Mazarin, France; 7 Medical Evidence Generation, Sanofi, 75008 Paris, France; 8 Global Medical Affairs Oncology, Sanofi, Cambridge, MA 02142, USA; 9 Vall d’Hebron University Hospital, Vall d’Hebron Institute of Oncology, 08035 Barcelona, Spain

**Keywords:** mCRPC, cabazitaxel, real-world, CUP, EAP, CAPRISTANA

## Abstract

We examined factors that may impact cabazitaxel treatment duration in a real-life setting in a compassionate use program, expanded access program, and prospective observational study in metastatic castration-resistant prostate cancer (mCRPC). Patients with mCRPC previously treated with docetaxel (N = 1621) received cabazitaxel 25 mg/m^2^ intravenously every 3 weeks until disease progression, death, unacceptable toxicity or physician/patient decision. The median number of cabazitaxel cycles was six (range, 1–49); 708 patients (43.7%) received >6 cycles. Patients receiving >6 cycles tended to have a better Eastern Cooperative Oncology Group performance status of 0–1 (*p* = 0.0017 for ≤6 vs. >6 cycles). Overall, 348 patients (21.5%) were ≥75 years of age; 139 (39.9%) received >6 cycles. The main reason for discontinuation was disease progression; however, in patients receiving 1–2 cycles, the main reason for discontinuation was adverse events. Only 52 patients (3.2%) progressed during cycles 1–2. Cabazitaxel was well tolerated in these studies, which included some elderly and frail patients, offering clinicians an important treatment option in the management of mCRPC. Proactive management of adverse events may allow patients to receive a higher number of cabazitaxel cycles and derive greater benefit.

## 1. Introduction

Prostate cancer is the second most commonly occurring male malignancy worldwide [1]. Early diagnosis is typically associated with a better prognosis. However, following castration-sensitive disease, approximately 10–20% of patients progress to a castration-resistant state [2]. In addition, approximately 5–21% of patients present with distant metastases at diagnosis [3,4].

In metastatic disease the standard treatment is surgical or chemical castration, and up to 85% of patients may respond initially [5,6]. However, nearly all patients will progress to metastatic castration-resistant prostate cancer (mCRPC) within 3 years of diagnosis [5,6,7,8].

In 2004, docetaxel was approved as a first-line treatment for mCRPC following the results of the pivotal TAX-327 Phase III trial, in which docetaxel demonstrated a significant improvement in overall survival compared with mitoxantrone [9]. Cabazitaxel is a second-generation taxane designed to overcome resistance to docetaxel and was selected for evaluation in clinical trials based on its activity in both docetaxel-sensitive and docetaxel-resistant cell lines [10]. Cabazitaxel was the first agent to demonstrate a survival benefit in the post-docetaxel setting and was approved as a second-line treatment for mCRPC in 2010 following the results of the Phase III TROPIC trial, where cabazitaxel was shown to confer a survival advantage vs. mitoxantrone (hazard ratio 0.70; 95% confidence interval: 0.59–0.83; *p* < 0.0001) [11].

However, the restrictive nature of clinical trial designs seldom accounts for patients with added complicating factors such as advanced age or comorbidities. As such, real-world studies provide the platform to corroborate the findings of large Phase III trials by incorporating more diverse patient cohorts and recognizing potential unmet medical needs in order to optimize patient care [12]. Following the positive results of TROPIC and the unmet medical need at the time, three studies were established to assess cabazitaxel in patients with mCRPC who had previously received docetaxel in a real-world setting, while providing early access to cabazitaxel in countries where commercial availability had not yet been established.

The cabazitaxel compassionate use program (CUP) [CABAZ_C_05005] and expanded access program (EAP) [NCT01254279] gave patients access to cabazitaxel for the management of post-docetaxel mCRPC and assessed the overall safety profile of cabazitaxel. CAPRISTANA (CABAZ_C_06092) was a prospective, observational study which evaluated the routine clinical use of cabazitaxel for the management of post-docetaxel mCRPC [13,14].

The objective of this current post hoc analysis was to examine the duration of cabazitaxel treatment, and factors that may impact cabazitaxel treatment duration, in a real-world setting.

## 2. Results

### 2.1. Patient Population

**Figure 1:**
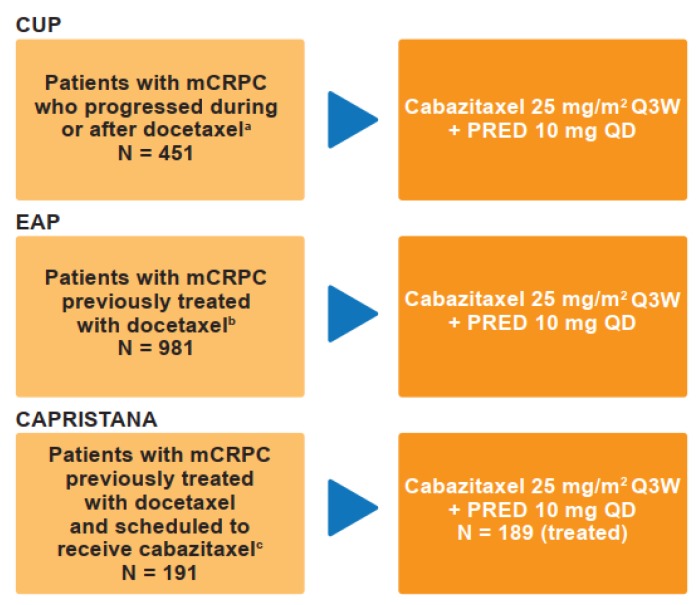
Study designs for the CUP, EAP and CAPRISTANA studies. ^a^ December 2010–May 2013; ^b^ July 2010–December 2014; ^c^ April 2012–June 2016. CUP: compassionate use program; EAP: expanded access program; mCRPC: metastatic castration-resistant prostate cancer; PRED: prednisone or prednisolone; Q3W: once every 3 weeks; QD: once daily.

A total of 1621 patients were available for analysis. In CUP and EAP, 1432 patients received cabazitaxel in routine clinical practice across 41 countries: in CUP, 451 patients across 12 countries received cabazitaxel between December 2010 and May 2013; in EAP, 981 patients across 29 countries received cabazitaxel between July 2010 and December 2014 [14]. In CAPRISTANA, 189 patients across six countries received cabazitaxel in routine clinical practice between April 2012 and June 2016 (Figure 1) [13].

### 2.2. Treatment Exposure

Across the total population of patients, the median number of cabazitaxel cycles received was six (range, 1–49) with 708 patients (43.7%) receiving >6 cycles and 913 (56.3%) receiving ≤6 cycles (Table 1). In total, 211 patients (13.0%) received ≥11 cycles of cabazitaxel; among these patients the median number of cabazitaxel cycles received was 14 (range, 11–49). Of note, in some countries included in the CUP/EAP/CAPRISTANA studies, patients did not receive >10 cabazitaxel cycles (10/47 countries; Luxembourg, Bangladesh, Peru, Singapore, Germany, India, Philippines, Poland, Serbia and Taiwan). For a list of the maximum number of cabazitaxel cycles received in each country, please see the online Supplementary Materials (Table S1).

### 2.3. Granulocyte-Colony Stimulating Factor (G-CSF) Use for Neutropenia

Prophylactic and therapeutic use of G-CSF was allowed at the physicians’ discretion. For the CUP and EAP studies, the protocols stated that primary prophylaxis with G-CSF (defined as use during Cycle 1; secondary prophylaxis defined as use in any subsequent cycle) should be considered in patients with high-risk clinical features that may predispose them to increased complications from prolonged neutropenia (≥65 years of age, poor performance status, previous episodes of febrile neutropenia, extensive prior radiation ports, poor nutritional status, or other serious comorbidities). G-CSF was administered during Cycle 1 in 54.2% of patients (Table 1).

### 2.4. Patient Characteristics That May Impact Cabazitaxel Treatment Duration

Baseline demographics and clinical characteristics for each treatment group defined by the number of cabazitaxel cycles received are summarized in Table 2, Table 3 and Table 4. This analysis investigated a large population of patients (N = 1621), with a number of patients presenting with aggressive disease characteristics at baseline. For example, lung metastases, liver metastases and other visceral/soft tissue metastases were present in 11.0%, 9.3% and 4.3% of patients at baseline, respectively, a short median time from mCRPC diagnosis was observed (1.7–1.8 years), and patients had received a high number of prior docetaxel cycles at last administration (medians ranging between 7 and 10 cycles) with the median cumulative dose of the last docetaxel administration ranging between 600 and 750 mg/m^2^.

Some baseline characteristics differed across the treatment groups defined according to the number of cabazitaxel cycles received, suggesting some baseline parameters may impact the duration of cabazitaxel treatment (≤6 vs. >6 cycles, Table 2; ≤4 vs. >4 cycles, Table 3; 1–2 vs. 3–10 vs. ≥11 cycles, Table 4), including Eastern Cooperative Oncology Group performance status (ECOG PS), frailty, number of metastatic sites (referring to organ systems), and prior docetaxel treatment duration. More patients in the treatment groups receiving fewer cabazitaxel cycles tended to have a poorer ECOG PS compared with the treatment groups that received more cabazitaxel cycles (Table 2, Table 3 and Table 4). In total, 21.5% of patients (348/1621) were ≥75 years of age and of these patients, 139 (40%) received >6 cabazitaxel cycles and 44 (12.6%) received ≥ 11 cabazitaxel cycles. Among frail patients (defined as patients with ECOG PS ≥2 and >75 years of age; n = 36), 13 patients (36.1%) received >6 cycles, two patients (5.6%) received ≥11 cycles and 14 patients (38.9%) received only 1–2 cycles of cabazitaxel. In multivariate analyses ECOG PS, the number of metastatic sites and prior exposure to docetaxel were all independently associated with the number of cabazitaxel treatment cycles received (Table S2).

### 2.5. Disease Progression during the First Two Cycles of Treatment

Across the CUP/EAP and CAPRISTANA studies, only 3.2% of patients (52/1621) progressed during the first two cycles of cabazitaxel treatment; of these patients, 19 had visceral metastases (lungs, liver, and other visceral/soft tissue) and 33 had other metastatic sites at baseline.

### 2.6. Reasons for Discontinuation According to Cabazitaxel Treatment Duration

**Figure 2:**
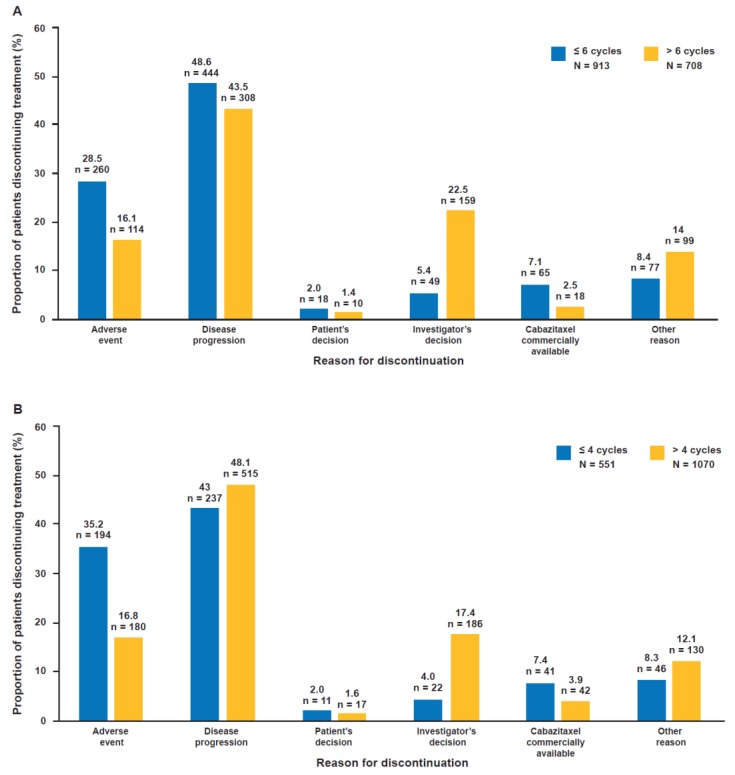

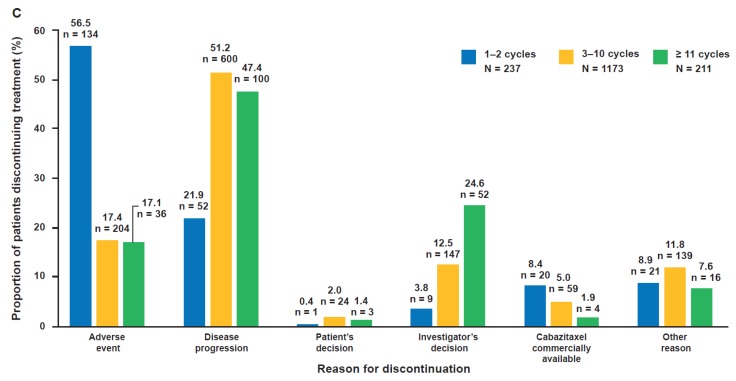
Reasons for treatment discontinuation according to the number of cabazitaxel treatment cycles received across the CUP/EAP and CAPRISTANA studies: (**A**) ≤6 cycles vs. >6 cycles; (**B**) ≤4 cycles vs. >4 cycles; (**C**) 1–2 cycles vs. 3–10 cycles vs. ≥11 cycles. CUP: compassionate use program; EAP: expanded access program.

The reasons for treatment discontinuation differed between patients receiving ≤6 vs. >6 cabazitaxel cycles, ≤4 vs. >4 cycles and 1–2 vs. 3–10 vs. ≥11 cycles (*p* < 0.0001) (Figure 2). The main reason for discontinuation was disease progression across all treatment groups defined by the number of cabazitaxel cycles received, with one exception: in patients receiving 1–2 cycles of cabazitaxel the main reason for discontinuation was adverse events (AEs). Patient request to discontinue treatment was low across all treatment groups and investigator decision to discontinue treatment increased as the treatment duration increased. Among frailer patients (ECOG PS ≥2 and >75 years of age; n = 36), AEs appeared to be the main reason for treatment discontinuation.

In patients receiving ≤6 (n = 23) vs. >6 (n = 13) cycles, 12 (52.2%) vs. 5 (38.5%) patients discontinued due to AEs and 7 (30.4%) vs. 3 (23.1%) patients discontinued due to disease progression, respectively. In patients receiving ≤4 (n = 18) vs. >4 (n = 18) cycles, 11 (61.1%) vs. 6 (33.3%) patients discontinued due to AEs and 4 (22.2%) vs. 6 (33.3%) patients discontinued due to disease progression, respectively. In patients receiving 1–2 (n = 14) vs. 3–10 (n = 20) vs. ≥11 (n = 2) cycles, 9 (64.3%) vs. 7 (35.0%) vs. 1 (50.0%) patient discontinued due to AEs and 3 (21.4%) vs. 7 (35.0%) vs. 0 patients discontinued due to disease progression, respectively.

## 3. Discussion

Cabazitaxel may offer clinicians a useful and important treatment option in the management of patients with mCRPC. This post hoc analysis of real-world data from the CUP/EAP and CAPRISTANA studies investigated a large, global patient population (N = 1621), with a number of patients presenting with aggressive disease characteristics at baseline. The patients enrolled in this pooled analysis were similar to the patient populations of the Phase III TROPIC and PROSELICA trials assessing cabazitaxel in patients with mCRPC post-docetaxel [11,15]. Cabazitaxel was well tolerated in this pooled analysis, which included a proportion of elderly patients, with many patients receiving a high number of treatment cycles. Some baseline characteristics (ECOG PS, frailty, metastatic sites, prior docetaxel treatment duration) may impact cabazitaxel treatment duration with the data suggesting that patients with a poorer prognosis, more aggressive disease or poor ECOG PS, tended to receive fewer cabazitaxel cycles. Multivariate analyses suggested that ECOG PS, number of metastatic sites and prior docetaxel treatment were all associated with cabazitaxel treatment duration.

During the first two cabazitaxel treatment cycles, the main reason for treatment discontinuation was AEs. However, during later cycles, more patients discontinued treatment due to disease progression. This may be due to a higher disease burden present at baseline and suggests that proactive management of AEs early in the course of cabazitaxel treatment may allow patients to go on to receive a higher number of cabazitaxel cycles and derive a greater benefit.

The CUP/EAP/CAPRISTANA studies included a proportion of patients that were elderly and/or frail (ECOG PS ≥2 and >75 years of age). Among the frailer patients 36% received >6 cycles and among patients ≥75 years of age, 40% received >6 cycles and 13% received > 0 cycles. Treating elderly patients in the clinic often represents a challenge due to age-related comorbidities and weakening organ function, therefore, chemotherapy is often avoided, even in the case of fit, elderly patients [16]. In mCRPC, elderly patients represent a significant proportion of patients being treated in clinical practice and they should have access to treatments that may prolong their survival and improve their outcome [16]. Notably, in the current analysis, amongst the fit, elderly patients (ECOG PS <2 and >75 years of age), 39% received >6 cycles and 12% received >10 cycles of cabazitaxel.

Elderly patients are more likely to experience certain AEs with cabazitaxel, particularly neutropenia and febrile neutropenia [17,18]. In the TROPIC and PROSELICA trials, the rates of neutropenia, fatigue, asthenia, pyrexia, dizziness, urinary tract infection, diarrhea and dehydration were higher amongst patients who were ≥65 years of age [11,15,18]. The main reason for treatment discontinuation in the elderly/frail patients in this analysis was AEs. However, with active AE management, elderly patients may be able to receive a greater number of cabazitaxel cycles, as shown in this post hoc analysis. No specific dose adjustment of cabazitaxel is recommended in elderly patients as no significant difference in cabazitaxel pharmacokinetics between patients <65 years (n = 100) and older (n = 70) has been observed. [17,18]. Furthermore, in the TROPIC study, there was no overall difference in cabazitaxel effectiveness between patients ≥65 years of age and younger [11,18]. Some studies have assessed, or are currently assessing, different cabazitaxel dosing schedules for elderly patients (e.g., weekly 8–10 mg/m^2^; twice-weekly 16 mg/m^2^ on Day 1 and 15 of a 4-week cycle) [19,20,21], however, dose modifications based on the age of the patient alone are not recommended [17,18]. An individual approach should be taken with management driven by a patient’s physiological age and functional status rather than their chronological age. [16] The International Society of Geriatric Oncology has developed guidelines for the treatment of elderly patients with prostate cancer which considers comorbidities, health status, dependence status, and nutritional status [16,22].

Other studies support this current post hoc analysis suggesting that cabazitaxel is well-tolerated in a real-world setting regardless of age [16,23,24,25,26]. From previous analyses of the CUP/EAP/CAPRISTANA data the most frequently reported treatment emergent AEs (TEAEs) were consistent with the safety profile of cabazitaxel reported in the TROPIC and PROSELICA studies [11,13,14,15]. Some studies have suggested that prophylactic use of G-CSF appears to be more frequent in elderly patients and may improve tolerability in elderly patients receiving cabazitaxel, particularly when given at Cycle 1 [16,27]. Prophylactic G-CSF was not allowed during Cycle 1 in the PROSELICA and TROPIC studies but was permitted in the CUP/EAP/CAPRISTANA studies; this may, in part, explain the lower rates of neutropenia observed in these studies [11,13,14,15]. This analysis showed that hematological AEs can be effectively managed and reduced when G-CSF is used.

Of note, at the time of the CUP/EAP/CAPRISTANA studies the recommended dosing for cabazitaxel was 25 mg/m^2^ every 3 weeks. In light of the PROSELICA data, where cabazitaxel 20 mg/m^2^ dosing was proven to be non-inferior to 25 mg/m^2^ in terms of overall survival, the Food and Drug Administration approved the lower dose of cabazitaxel for the treatment of mCRPC, with 25 mg/m^2^ recommended for select patients at the physician’s discretion [15,18].

It is important to note that the CUP, EAP and CAPRISTANA studies are associated with certain limitations. As the studies were conducted across many countries, the impact of regional variation should be considered. For example, in some countries, longer treatment duration and a higher number of treatment cycles were considered routine practice and undertaken more often than in other countries. In addition, variation in regional guidelines for the prophylactic and therapeutic use of G-CSF for neutropenia may have influenced safety outcomes. Furthermore, there will have been variability among the sites regarding use of electronic records, which could potentially lead to missing data and incomplete representation of cabazitaxel use in the clinical setting. Finally, the median time from mCRPC diagnosis currently experienced in clinical practice is potentially longer than when these studies were undertaken due to the availability of new antiandrogen treatments, such as abiraterone and enzalutamide, which were not utilized at the time of these studies and are now being used earlier in the course of the disease.

In patients with mCRPC who have received prior docetaxel, treatment options include cabazitaxel, abiraterone or enzalutamide, which have all proven effective in the post-docetaxel setting [11,28,29]. With no head-to-head comparisons for these agents, no predictive biomarkers in everyday clinical practice to guide treatment options, and no defined optimal treatment sequence, treatment decisions in the real-world are often based on disease behavior (progression and response to treatment), comorbidities, performance status, AE profiles of the treatments and patient preference. If patients do not respond to one hormonal therapy, then they may benefit from receiving cabazitaxel rather than an alternative hormonal agent. In the recent CARD study cabazitaxel significantly improved overall survival and other clinical outcomes over abiraterone or enzalutamide, with an acceptable safety profile, when given to patients with mCRPC who had previously received docetaxel and had progression with 12 months of treatment with the alternative hormonal agent (abiraterone or enzalutamide) [30]. Abiraterone and enzalutamide are often given after docetaxel, instead of cabazitaxel, because of the differing AE profile. In light of the CARD results, this post hoc analysis shows in a real-world clinical practice setting, across a large patient population, that cabazitaxel is well tolerated with associated AEs managed effectively and can be used in the elderly and frail patient populations.

In the pivotal cabazitaxel study, TROPIC, cabazitaxel improved survival and other clinical outcomes compared with mitoxantrone, despite the fact that 72% of patients receiving cabazitaxel had docetaxel-resistant disease (disease progression during prior docetaxel treatment or within 3 months of the last docetaxel dose) [11]. Cabazitaxel offers the chance for a response in patients with mCRPC who are resistant, or develop resistance to docetaxel.

Another field of interest for taxanes including cabazitaxel is in cancer that has an unknown primary origin. In a recent study (CUPISCO), analysis of 303 tissue samples with an unknown primary cause revealed that 32% could be treated with a molecularly targeted therapy [31]. However, in another study (GEFCAPI 04), where gene expression analysis was used to identify the most likely primary tumor, the best available targeted therapy and other treatments tailored to the primary tumor failed to improve disease progression or survival compared with chemotherapy [32]. For cancers of unknown primary origin taxanes are recognized as a chemotherapeutic option, so cabazitaxel could be a useful treatment option to be studied in these patients that have resistance to docetaxel [33,34]. Furthermore, it has been shown that patients with some specific subtypes of prostate cancer, such as luminal B (classified using gene expression signatures) experience significant improvements in time to castration resistance and overall survival following treatment with docetaxel plus androgen deprivation therapy (ADT) [35]. This biomarker analysis suggested that a novel genetic signature could be used to inform treatment decisions and select patients that may have improved responses to chemotherapy. In patients with the luminal B subtype who progress during or after treatment with docetaxel plus ADT, cabazitaxel could be an alternative therapeutic option.

## 4. Materials and Methods

### 4.1. Study Designs and Treatment Protocol

The study designs for CUP, EAP and CAPRISTANA are described in Figure 1. CUP, EAP and CAPRISTANA were international, multicenter, prospective registry studies. Across all studies, patients were scheduled to receive cabazitaxel 25 mg/m^2^ intravenously every 3 weeks plus oral prednisone or prednisolone 10 mg daily. In the CUP and EAP studies, cabazitaxel was administered until disease progression, death, unacceptable toxicity, physician/patient decision, or the commercial availability of cabazitaxel (in some countries); in CAPRISTANA, patients were followed until death or up to 1.5 years (whichever came first) after initiation of cabazitaxel. In certain countries, the commercial availability of cabazitaxel, and also the availability of new treatments, may have resulted in the early discontinuation of cabazitaxel and removal of patients from the CUP and EAP studies. Study designs and treatment protocols have been described in detail previously for CUP, EAP and CAPRISTANA in their respective primary publications [13,14].

### 4.2. Patient Population

Key inclusion criteria across the studies included patients ≥18 years of age with mCRPC, previously treated with docetaxel, and scheduled to receive cabazitaxel. In addition, the CUP and EAP studies included patients that had a life expectancy of >3 months, an ECOG PS score of ≤2, and adequate bone marrow, liver and renal function. The studies were conducted in accordance with the principles outlined in the Declaration of Helsinki (18th World Medical Assembly, 1964) and all its subsequent amendments. Each patient provided signed, written, informed consent before enrolment. Standard patient and disease characteristics, and treatment history were collected at baseline. For further details surrounding the inclusion and exclusion criteria for each trial, please see the previous publications [13,14].

### 4.3. Study Objectives and Endpoints

The primary objective of the CUP and EAP studies was to allow access to cabazitaxel before its commercial availability in patients with mCRPC whose disease had progressed during or after docetaxel treatment; secondary objectives included assessment of the overall safety of cabazitaxel and in some countries health-related quality of life data were also collected. The primary objective of the CAPRISTANA study was to observe usage patterns of cabazitaxel; secondary objectives included further description of cabazitaxel usage patterns including subsequent therapies and use of G-CSF, clinical outcomes, health-related quality of life and safety. Results for the primary and secondary objectives of the CUP, EAP and CAPRISTANA studies have been previously published [13,14]. This manuscript presents a post hoc analysis with the objective of assessing the duration of cabazitaxel treatment in a real-world setting using data from the three registry studies.

### 4.4. Safety Assessments

Safety assessments included analysis of TEAEs and serious AEs (SAEs). A TEAE was defined as any untoward medical event or AE occurring or worsening during the on-treatment period (from the first administration of cabazitaxel to ≤30 days after the last administration of cabazitaxel). A SAE was defined as any untoward medical occurrence or AE that resulted in death, was life threatening, required hospitalization, or resulted in persistent or significant disability/incapacity. AEs were graded according to the National Cancer Institute Common Terminology Criteria for Adverse Events, version 4.0 [36].

### 4.5. Statistical Analyses

Patient baseline characteristics, reasons for treatment discontinuation and disease progression were assessed according to cabazitaxel treatment duration (≤median [6] vs. >median [6] cycles, ≤4 vs. >4 cycles and 1–2 vs. 3–10 vs. ≥11 cycles); *P* values were provided for comparisons between the different treatment groups (based on the number of cabazitaxel cycles received), using the Chi-Square test, Fisher’s Exact test, Wilcoxon Mann–Whitney test or Kruskal–Wallis test. Odds ratios and confidence intervals were based on a logistic regression analysis.

### 4.6. Cabazitaxel Treatment Cycles

To assess cabazitaxel treatment duration and factors that may impact treatment duration in this post hoc analysis, subgroups of patients were defined and assessed according to the number of cabazitaxel treatment cycles they received: ≤6 vs. >6 cycles; ≤4 vs. >4 cycles; 1–2 vs. 3–10 vs. ≥11 cycles. A cut-off of six cabazitaxel cycles were chosen because this was the median number of cabazitaxel cycles received in both the CUP/EAP and CAPRISTANA studies. Additionally, the identification and assessment of the subset of patients who discontinued cabazitaxel after ≤2 cycles was of interest, as it was hypothesized that their main reason for cabazitaxel treatment discontinuation would most likely be due to tolerability issues. Incorporating the analysis of patients who discontinued after ≤4 treatment cycles would therefore capture those patients discontinuing due to AEs but also those patients not responding to cabazitaxel treatment. Finally, the subset of patients who had a long duration of cabazitaxel treatment (i.e., beyond 10 cycles), were identified to assess whether any particular factors attributed to longer treatment duration.

## 5. Conclusions

This global, real-world post hoc analysis of pooled data from the CUP/EAP and CAPRISTANA studies further demonstrates the tolerability profile of cabazitaxel in a large population of patients and supports the use of cabazitaxel in patients with mCRPC after docetaxel, including patients who are elderly and/or frail. Some baseline characteristics may impact cabazitaxel treatment duration and discontinuation, and identification of these factors may help inform treatment decisions. Proactive and active management of AEs early in the course of cabazitaxel treatment may allow patients to receive a higher number of cabazitaxel cycles and derive a greater benefit.

## Figures and Tables

**Table 1:** Cabazitaxel treatment and granulocyte-colony stimulating factor (G-CSF) use according to the number of cabazitaxel treatment cycles received across the CUP/EAP and CAPRISTANA studies.

Treatment Characteristics	CUP/EAP/CAPRISTANA			
	N = 1621			
	Cabazitaxel Cycles Received			
	≤6 n = 913		>6 n = 708	
Median cabazitaxel cycles, n (range)	4 (1–6)		10 (7–49)	
Median duration of cabazitaxel exposure, months (range)	2.8 (1–6)		6.9 (5–35)	
G-CSF during Cycle 1, n (%) Prophylactic Therapeutic Both	n = 499 (54.7) 385 (42.2) 69 (7.6) 45 (4.9)		n = 380 (53.7) 314 (44.4) 33 (4.7) 33 (4.7)	
	**Cabazitaxel cycles received**			
	**≤4** **n = 551**		**>4** **n = 1070**	
Median cabazitaxel cycles, n (range)	3 (1–4)		8 (5–49)	
Median duration of cabazitaxel exposure, months (range)	2.1 (1–5)		5.8 (3–35)	
G-CSF during Cycle 1, n (%) Prophylactic Therapeutic Both	n = 297 (53.9) 224 (40.7) 43 (7.8) 30 (5.4)		n = 582 (54.4) 475 (44.4) 59 (5.5) 48 (4.5)	
	**Cabazitaxel cycles received**			
	**1–2** **n = 237**	**3–10** **n = 1173**		**≥11** **n = 211**
Median cabazitaxel cycles, n (range)	1 (1–2)	6 (3–10)		14 (11–49)
Median duration of cabazitaxel exposure, months (range)	0.7 (1–2)	4.3 (2–11)		10.3 (8–35)
G-CSF during Cycle 1, n (%) Prophylactic Therapeutic Both	n = 141 (59.5) 100 (42.2) 25 (10.5) 16 (6.8)	n = 632 (53.9) 515 (43.9) 66 (5.6) 51 (4.3)		n = 106 (50.2) 84 (39.8) 11 (5.2) 11 (5.2)

CUP: compassionate use program; EAP: expanded access program; G-CSF: granulocyte-colony stimulating factor.

**Table 2:** Patient baseline characteristics according to the number of cabazitaxel treatment cycles received (≤6 vs. >6 cycles) across the CUP/EAP and CAPRISTANA studies.

Patient Baseline Characteristics	CUP/EAP/CAPRISTANA N = 1621		
	Cabazitaxel Cycles Received		
	≤6 n = 913	>6 n = 708	*p* Value
Median age, years (range)	68.0 (42–89)	68.0 (43–89)	0.0855
Age, n (%) <65 years 65–75 years ≥75 years	271 (29.7) 433 (47.4) 209 (22.9)	230 (32.5) 339 (47.9) 139 (19.6)	0.2227
ECOG PS, n (%) 0–1 2 ^a^	n = 912 816 (89.5) 96 (10.5)	n = 708 665 (93.9) 43 (6.1)	0.0015
Frailty, n (%) ECOG PS <2 and ≤75 years ECOG PS <2 and >75 years ECOG PS ≥2 and ≤75 years ECOG PS ≥2 and >75 years	n = 912 660 (72.4) 156 (17.1) 73 (8.0) 23 (2.5)	n = 708 566 (79.9) 99 (14.0) 30 (4.2) 13 (1.8)	0.0016
Median time from prostate cancer diagnosis, years (range)	4.5 (0–22)	4.7 (0–20)	0.0659
Median time from mCRPC diagnosis, years (range)	1.7 (0–14)	1.8 (0–12)	0.1298
Median docetaxel cycles at last administration, n (range)	7 (1–69)	8 (1–58)	<0.0001
Median cumulative dose of last docetaxel administration, mg/m^2^ (range)	600 (50–5145)	675 (105–8700)	0.0005
Metastatic sites, n (%) Bone Regional lymph nodes Lungs Liver Visceral, other soft tissue	n = 912 829 (90.8) 282 (30.9) 111 (12.2) 105 (11.5) 47 (5.1)	n = 707 630 (89.0) 214 (30.2) 68 (9.6) 46 (6.5) 23 (3.2)	-
Number of metastatic sites, n (%) 0 1 ≥2	n = 913 1 (0.1) 298 (32.6) 614 (67.3)	n = 708 1 (0.1) 272 (38.4) 435 (61.4)	0.0254
Pain at baseline (CAPRISTANA study only), n (%) None Moderate Severe	n = 86 15 (17.4) 63 (73.3) 8 (9.3)	n = 68 18 (26.5) 47 (69.1) 3 (4.4)	0.2457

^a^ Includes one patient with ECOG PS 3 receiving ≤6 cabazitaxel cycles. CUP: compassionate use program; EAP: expanded access program; ECOG PS: Eastern Cooperative Oncology Group performance status; mCRPC: metastatic castration-resistant prostate cancer.

**Table 3:** Patient baseline characteristics according to the number of cabazitaxel treatment cycles received (≤4 vs. >4 cycles) across the CUP/EAP and CAPRISTANA studies.

Patient Baseline Characteristics	CUP/EAP/CAPRISTANA N = 1621		
	Cabazitaxel Cycles Received		
	≤4 n = 551	>4 n = 1070	*p* Value
Median age, years (range)	69.0 (42–89)	68.0 (42–89)	0.2033
Age, n (%) < 65 years 65–75 years ≥ 75 years	165 (29.9) 258 (46.8) 128 (23.2)	336 (31.4) 514 (48.0) 220 (20.6)	0.4562
ECOG PS, n (%) 0–1 2 ^a^	n = 550 476 (86.5) 74 (13.5)	n = 1070 1005 (93.9) 65 (6.1)	<0.0001
Frailty, n (%) ECOG PS <2 and ≤75 years ECOG PS <2 and >75 years ECOG PS ≥2 and ≤75 years ECOG PS ≥2 and >75 years	n = 550 386 (70.2) 90 (16.4) 56 (10.2) 18 (3.3)	n = 1070 840 (78.5) 165 (15.4) 47 (4.4) 18 (1.7)	<0.0001
Median time from prostate cancer diagnosis, years (range)	4.6 (0–22)	4.7 (0–20)	0.1603
Median time from mCRPC diagnosis, years (range)	1.7 (0–11)	1.8 (0–14)	0.1359
Median docetaxel cycles at last administration, n (range)	7 (1–69)	8 (1–68)	0.0061
Median cumulative dose of last docetaxel administration, mg/m^2^ (range)	600 (50–5145)	675 (50–8700)	0.0063
Metastatic sites, n (%) Bone Regional lymph nodes Lungs Liver Visceral, other soft tissue	n = 550 497 (90.2) 173 (31.4) 80 (14.5) 75 (13.6) 29 (5.3)	n = 106 962 (89.9) 323 (30.2) 99 (9.3) 76 (7.1) 41 (3.8)	-
Number of metastatic sites, n (%) 0 1 ≥2	n = 551 1 (0.2) 171 (31.0) 379 (68.8)	n = 1070 1 (<0.1) 399 (37.3) 670 (62.6)	0.0190
Pain at baseline (CAPRISTANA study only), n (%) None Moderate Severe	n = 48 6 (12.5) 36 (75.0) 6 (12.5)	n = 106 27 (25.5) 74 (69.8) 5 (4.7)	0.0737

^a^ Includes one patient with ECOG PS 3 receiving ≤4 cabazitaxel cycles. CUP: compassionate use program; EAP: expanded access program; ECOG PS: Eastern Cooperative Oncology Group performance status; mCRPC: metastatic castration-resistant prostate cancer.

**Table 4:** Patient baseline characteristics according to the number of cabazitaxel treatment cycles received (1–2 vs. 3–10 vs. ≥11 cycles) across the CUP/EAP and CAPRISTANA studies.

Patient Baseline Characteristics	CUP/EAP/CAPRISTANA N = 1621			
	Cabazitaxel Cycles Received			
	1–2 n = 237	3–10 n = 1173	≥11 n = 211	*p* Value
Median age, years (range)	70.0 (42–89)	68.0 (42–89)	68.0 (49–87)	0.0114
Age, n (%) <65 years 65–75 years ≥75 years	64 (27.0) 103 (43.5) 70 (29.5)	377 (32.1) 562 (47.9) 234 (19.9)	60 (28.4) 107 (50.7) 44 (20.9)	0.0177
ECOG PS, n (%) 0–1 2 ^a^	n = 237 197 (83.1) 40 (16.9)	n = 1172 1082 (92.3) 90 (7.7)	n = 211 202 (95.7) 9 (4.3)	0.0001
Frailty, n (%) ECOG PS <2 and ≤75 years ECOG PS <2 and >75 years ECOG PS ≥2 and ≤75 years ECOG PS ≥2 and >75 years	n = 237 149 (62.9) 48 (20.3) 26 (11.0) 14 (5.9)	n = 1172 906 (77.3) 176 (15.0) 70 (6.0) 20 (1.7)	n = 211 171 (81.0) 31 (14.7) 7 (3.3) 2 (0.9)	<0.0001
Median time from prostate cancer diagnosis, years (range)	4.9 (1–22)	4.4 (0–20)	5.5 (1–18)	0.0002
Median time from mCRPC diagnosis, years (range)	1.8 (0–10)	1.7 (0–14)	1.8 (0–10)	0.2483
Median docetaxel cycles at last administration, n (range)	8 (1–34)	8 (1–69)	10 (2–49)	<0.0001
Median cumulative dose of last docetaxel administration, mg/m^2^ (range)	600 (50–2850)	610.6 (50–8700)	750 (120–2830)	<0.0123
Metastatic sites, n (%) Bone Regional lymph nodes Lungs Liver Visceral, other soft tissue	n = 236 211 (89.0) 75 (31.6) 42 (17.7) 39 (16.5) 9 (3.8)	n = 1173 1062 (90.5) 361 (30.8) 119 (10.1) 101 (8.6) 54 (4.6)	n = 210 186 (88.2) 60 (28.4) 18 (8.5) 11 (5.2) 7 (3.3)	-
Number of metastatic sites, n (%) 0 1 ≥2	n = 237 1 (0.4) 71 (30.0) 165 (69.6)	n = 1173 0 415 (35.4) 758 (64.6)	n = 211 1 (0.5) 84 (39.8) 126 (59.7)	0.0203
Pain at baseline (CAPRISTANA study only), n (%) None Moderate Severe	n = 8 3 (37.5) 4 (50.0) 1 (12.5)	n = 127 24 (18.9) 93 (73.2) 10 (7.9)	n = 19 6 (31.6) 13 (68.4) 0	0.2260

^a^ Includes one patient with ECOG PS 3 receiving 1–2 cabazitaxel cycles. CUP: compassionate use program; EAP: expanded access program; ECOG PS: Eastern Cooperative Oncology Group performance status; mCRPC: metastatic castration-resistant prostate cancer.

## Supplementary Materials

The following are available online at https://www.mdpi.com/2072-6694/12/4/995/s1, Table S1. Maximum number of cabazitaxel cycles received by patients in each country included in the CUP, EAP and CAPRISTANA studies and Table S2. Multivariate analysis of baseline characteristics associated with cabazitaxel treatment duration.
