# Supplementary material for: Effect of Baseline Characteristics on Cabazitaxel Treatment Duration in Patients with Metastatic Castration-Resistant Prostate Cancer: A Post Hoc Analysis of the Compassionate Use/Expanded Access Programs and CAPRISTANA Registry
Source: Cancers (Basel). 2020 Apr 17;12(4):995. doi: 10.3390/cancers12040995 (PMC7226228; doi:10.3390/cancers12040995)
Supplement: Supplementary file 1 [file cancers-12-00995-s001.pdf]

cancers

MDPI

Article

Effect of Baseline Characteristics on Cabazitaxel Treatment Duration in Patients with Metastatic Castration-Resistant Prostate Cancer: A Post Hoc Analysis of the Compassionate Use/Expanded Access Programs and CAPRISTANA Registry

Zafar Malik, Giuseppe Di Lorenzo, Angelika Pichler, Ugo De Giorgi, Simon Hitier, Evelyne Ecstein-Fraisse, Ayse Ozatilgan and Joan Carles

Online supplementary material

Table S1. Maximum number of cabazitaxel cycles received by patients in each country included in the CUP, EAP and CAPRISTANA studies.

Registry study	Country	Maximum number of cabazitaxel cycles
EAP	Luxembourg	4
CUP	Bangladesh	6
CUP	Peru	6
EAP	Singapore	9
CUP	Germany	10
EAP	India	10
EAP	Philippines	10
EAP	Poland	10
EAP	Serbia	10
EAP	Taiwan	10
EAP	Hungary	11
EAP	Sweden	11
CAPRISTANA	Czech Republic	12
CAPRISTANA	Russian Federation	12
EAP	Belgium	12
EAP	Denmark	12
CAPRISTANA	Austria	13
CUP	Thailand	13
EAP	Austria	13
EAP	Ireland	14
EAP	United Kingdom	16
EAP	Bosnia and Herzegovina	17
CUP	Greece	19
EAP	Portugal	19
EAP	Czech Republic	20
EAP	Finland	20
CAPRISTANA	Bulgaria	21
CUP	Netherlands	21
EAP	Bulgaria	21
CUP	Brazil	22
CUP	Turkey	22
CUP	Republic of Korea	23
CAPRISTANA	Lebanon	24
CUP	Lebanon	24

EAP	Croatia	24
CUP	Slovenia	25
CAPRISTANA	Spain	26
EAP	Kazakhstan	26
EAP	Mexico	26
EAP	Slovakia	26
EAP	Spain	26
EAP	Canada	27
EAP	Australia	33
EAP	Italy	33
CUP	Norway	34
EAP	Malaysia	37
EAP	Romania	49

CUP: compassionate use program; EAP: expanded access program.

Table S2. Multivariate analysis of baseline characteristics associated with cabazitaxel treatment duration**.**

Factor	Modalities	*p* value	Odds ratio (95% CI)
**> 6 vs. ≤ 6 cabazitaxel treatment cycles**			
ECOG PS	≥ 2 vs. 0–1	0.0090	1.72 (1.14–2.58)
Number of metastatic sites	≥ 2 vs. 0–1	0.0500	1.25 (1.00–1.56)
Cumulative dose of last docetaxel administration, mg/m²	225–450 vs. < 225	0.1510	0.67 (0.38–1.16)
	450–675 vs. < 225	0.5032	0.84 (0.49–1.41)
	675–900 vs. < 225	0.0231	0.54 (0.31–0.92)
	≥ 900 vs. < 225	0.0136	0.52 (0.30–0.87)
**> 4 vs. ≤ 4 cabazitaxel treatment cycles**			
ECOG PS	≥ 2 vs. 0–1	0.0001	2.14 (1.47–3.12)
Number of metastatic sites	≥ 2 vs. 0–1	0.0409	1.28 (1.01–1.61)
**≥ 11 vs. 3–10 vs. 1–2 cabazitaxel treatment cycles**			
ECOG PS	≥ 2 vs. 0–1	0.0001	2.21 (1.48–3.31)
Number of metastatic sites	≥ 2 vs. 0–1	0.0364	1.30 (1.02–1.66)
Total number of prior docetaxel cycles	-	0.0104	0.98 (0.96–0.99)

Multivariate logistic regression was performed to identify baseline characteristics and factors associated with the number of cabazitaxel cycles received. The following factors were entered into the model: age (years), ECOG PS (≥ 2 vs. 0–1), time from prostate cancer diagnosis (years), time from mCRPC diagnosis (years), prior docetaxel cycles at last administration, cumulative dose of last prior docetaxel administration (mg/m²; 225–450, 450–675, 675–900, ≥ 900 vs. < 225), and number of metastatic sites (≥ 2 vs. 0–1). Selection of statistically associated factors was done using a stepwise approach (with an entry threshold of 20% and a stay threshold of 5%). For the analysis assessing > 6 vs. ≤ 6 and > 4 vs. ≤ 4 cabazitaxel cycles, odds ratios were provided considering the lower number of cabazitaxel cycles as the reference. For the analysis assessing ≥ 11 vs. 3–10 vs. 1–2 cabazitaxel cycles an ordinal multinomial logistic regression was used to identify associated factors using the same approach as described above. CI: confidence interval; ECOG PS: Eastern Cooperative Oncology Group performance status.
